# 

**Published:** 1907-12

**Authors:** 


### CONTENTS.

ORIGINAL COMMUNICATIONS—

Curative Treatment of Puerperal Eclampsia, By Joseph Eve Allen, Augusta, Ga .......... 519
Cholecystitis, With Special Reference to Etiology and Diagnosis, By T. E. Potter, M. D., St. Joseph, Mo. 524
Relations of the Doctor to the Alcoholic Problem By T. D. Crothers, Hartford, Conn .......... 529
The Physician and the Venereal Peril, By Stewart R. Roberts, M. S., M. D., Professor of Physiology, Atlanta School of Medicine, Atlanta, Ga .......... 538
Special Meeting of the Fulton County Medical Society for the Disucssion of theSocial Evil. Report of Committee on Sanitary and Prophylaxis .......... 542
Pneumatic Scrotal Compressor for Use In Epididymitis, By Edgar Ballenger, M. D., Lecturer on Genito-Urinary Diseases, Atlanta School of Medicine, Atlanta, Ga .......... 561
"Report of One Hundred and Eleven Cases of Cancer Treated With the X-Ray With Comments Thereon," By Dr. Ennion G. Williams .......... 566
Baseball as a Safety Valve .......... 570

EDITORIAL NOTES AND COMMENTS—

Book Reviews .......... 578

Medical 1tems .......... 575

